# Suppressing Respiration Effects when Geometric Distortion Is Corrected Dynamically by Phase Labeling for Additional Coordinate Encoding (PLACE) during Functional MRI

**DOI:** 10.1371/journal.pone.0156750

**Published:** 2016-06-03

**Authors:** Zahra Faraji-Dana, Fred Tam, J. Jean Chen, Simon J. Graham

**Affiliations:** 1 Department of Medical Biophysics, University of Toronto, Toronto, Canada; 2 Sunnybrook Research Institute, Sunnybrook Health Sciences Centre, Toronto, Canada; 3 Rotman Research Institute, Baycrest Health Sciences Centre, Toronto, Canada; Institute of Psychology, Chinese Academy of Sciences, CHINA

## Abstract

Echo planar imaging (EPI) suffers from geometric distortions caused by magnetic field inhomogeneities, which can be time-varying as a result of small amounts of head motion that occur over seconds and minutes during fMRI experiments, also known as “dynamic geometric distortion”. Phase Labeling for Additional Coordinate Encoding (PLACE) is a promising technique for geometric distortion correction without reduced temporal resolution and in principle can be used to correct for motion-induced dynamic geometric distortion. PLACE requires at least two EPI images of the same anatomy that are ideally acquired with no variation in the magnetic field inhomogeneities. However, head motion and lung ventilation during the respiratory cycle can cause changes in magnetic field inhomogeneities within the EPI pair used for PLACE. In this work, we exploited dynamic off-resonance in k-space (DORK) and averaging to correct the within EPI pair magnetic field inhomogeneities; and hence proposed a combined technique (DORK+PLACE+averaging) to mitigate dynamic geometric distortion in EPI-based fMRI while preserving the temporal resolution. The performance of the combined DORK, PLACE and averaging technique was characterized through several imaging experiments involving test phantoms and six healthy adult volunteers. Phantom data illustrate reduced temporal standard deviation of fMRI signal intensities after use of combined dynamic PLACE, DORK and averaging compared to the standard processing and static geometric distortion correction. The combined technique also substantially improved the temporal standard deviation and activation maps obtained from human fMRI data in comparison to the results obtained by standard processing and static geometric distortion correction, highlighting the utility of the approach.

## Introduction

Functional magnetic resonance imaging (fMRI) methods typically record signal intensity changes based on hemodynamic responses that accompany neuronal activity, through the blood oxygenation level dependent (BOLD) effect [1–3]. As BOLD responses evolve on the timescale of seconds, spatial encoding must be conducted much more rapidly than in conventional anatomical MRI. The majority of fMRI studies employ single-shot echo planar imaging (EPI) [4] which, through the use of a raster scan k-space trajectory, typically enables spatial encoding of a single slice in less than 100 ms, and multislice whole-brain coverage in 1- 2s. Although EPI enables fMRI with adequate temporal resolution, the raster scan provides much more rapid data acquisition in the frequency encoding direction (k_x_) than in the phase encoding (PE) (k_y_) direction. This imbalance in temporal sampling enhances sensitivity to magnetic field inhomogeneity produced by spatial variations in magnetic susceptibility, particularly at air-tissue interfaces [5][6], which cannot be completely suppressed using the conventional static shimming procedures available on whole-body MRI systems. The resulting characteristic EPI artifacts include geometric distortion (localized "compression" or "stretching" of MRI signals in the PE direction) and signal loss (typically near air-filled sinuses) [7–9].

Focusing on geometric distortion, a number of methods have been introduced to correct EPI data by first mapping the estimated magnetic field inhomogeneities, then using the field maps to correct for distortions in image post-processing [5,6,10–19]. Field maps can be produced by various means, such as by subtracting the unwrapped phase images acquired at different echo times [5][6], using multi-channel modulations [10], performing point spread function mapping [11][12] and reversing gradient polarities [13]. In these approaches, preliminary scans are undertaken to generate the estimated field maps that are consequently applied to all images in the EPI time series.

Although beneficial, these approaches may be insufficient in many situations. It is well known that involuntary head motion is present during fMRI in variable amounts depending on the experiment (e.g., study of task-related or resting state brain activity) and the individual that is imaged (e.g., ranging from compliant young healthy adults, to challenging patient populations with cognitive or motor impairments). Among the problems introduced by head motion, such as the partial volume effects induced by tissue misalignment [20] and spin history artifacts [21], slight shifts in the location and orientation of the brain with respect to the main magnetic field produce time-varying magnetic field inhomogeneities, and thus non-rigid “dynamic geometric distortions” in EPI time series data [14,15,17]. Depending on the amount of motion present and the static magnetic field strength of the MRI system, use of a single field map acquired immediately prior to fMRI data collection (subsequently referred to as a “static” field map) may be insufficient to correct dynamic geometric distortions.

Several approaches have been developed to obtain dynamic field maps, as a consequence. One approach is dual echo time EPI [14], however the associated time penalty is a limitation. Another is to predict dynamic field maps using rigid-body motion parameters, but this approach depends on the accuracy of head motion estimates (themselves potentially corrupted by geometric distortion)[15], and on the accuracy of magnetic susceptibility estimates within the head [16]. The use of relative field maps has also been proposed, whereby dynamic maps are estimated by subtracting the unwrapped phase images in the time series from a reference image [17]. Similar to some of the static field map correction methods mentioned above [5][6], this approach requires phase unwrapping. Robust phase unwrapping is difficult to achieve, however, especially in the presence of strong magnetic susceptibility or chemical shift effects [5]. Iterative optimization algorithms provide another alternative, whereby the undistorted image and the field map are reconstructed at each point in time using spiral-in/ spiral-out k-space trajectories [18], but with substantially increased computational complexity.

A promising method that overcomes some of the limitations of these recent studies [5,6,10–18] is known as phase labeling for additional coordinate encoding (PLACE) [19]. The PLACE method requires at least two EPI acquisitions of the same anatomy, with one acquisition using a k-space raster shifted in the k_y_ direction by one PE increment. The resulting phase ramp between the two images encodes the original spatial coordinate of the signal, which can then be retrieved by simple phase calculations without phase unwrapping. The PLACE method has shown promise in correcting static geometric distortions (termed “sPLACE”) equally well as conventional field mapping approaches [22,23]. In principle, PLACE can also be used to correct for dynamic geometric distortion (termed “dPLACE”) by successively calculating new PLACE maps from EPI pairs throughout time series data collection.

The utility of dPLACE depends on the assumption that there is a negligible change in magnetic field inhomogeneity between EPI pairs. However, in fMRI experiments, dynamic magnetic field inhomogeneities can arise from EPI time point to time point not only from between the EPI images can arise from head motion, but also from lung ventilation effects during the respiratory cycle. The latter effects are a known source of spurious phase ramps in the k_y_ direction, which can cause subtle, corresponding spatial shifts in a EPI given slice location [24–26]. Thus, a robust implementation of dPLACE will require additional correction strategies to be adopted.

Pfeuffer *et al*. [25] performed resting-state fMRI of two healthy young adults and showed that respiratory artifacts were well corrected using a method known as dynamic off-resonance in k-space (DORK) [27]. This method employs the phase from a navigator echo and the centre of the raster scan in k-space to estimate off-resonance effects, and then applies appropriate phase-ramp corrections. Zeller *et al*. performed additional related work in seven healthy young adults without mapping brain activity [26], reporting more pronounced off-resonance effects observed inferiorly in the brain (closer to the lungs), and that PLACE can be improved by temporal averaging or by using DORK. The DORK-based approach has the advantage of preserving temporal resolution, whereas temporal averaging over the entire time series data collection results in a static field map that suppresses the dynamic field fluctuations of interest.

These initial studies [25,26] suggest, but do not establish conclusively, that a combined approach involving DORK and dPLACE might be beneficial for fMRI. This assertion needs to be investigated directly. The respiratory correction provided by DORK has a level of experimental error and it is unclear whether the combination of both techniques reduces dynamic geometric distortion sufficiently to improve fMRI results in a practical application. Furthermore, it should be possible to perform more sophisticated temporal averaging to suppress the experimental uncertainty in DORK corrections, recognizing that field maps acquired at the same head position should be highly similar. The present work is consequently designed to investigate these lines of thought. It is hypothesized that DORK combined with dPLACE and temporal averaging of PLACE maps acquired at the same head position improves image stability and statistical inference of brain activity in fMRI of young healthy adults. The experimental findings are subsequently discussed in the context of whether combining DORK, dPLACE and temporal averaging of PLACE maps provides a robust and comprehensive solution for suppressing the effects of dynamic geometric distortion in fMRI studies.

## Materials and Methods

The study was undertaken through successive stages of technical development, initial testing and validation in phantoms, and subsequent human fMRI experiments. The scientific methodology for each component is described in detail below.

### Technical Development

#### Phase Labeling for Additional Coordinate Encoding (PLACE)

Pulse sequence modifications were undertaken to enable dPLACE during standard multi-slice single-shot EPI on a research-dedicated MRI system operating at 3 T (Trio with Total Imaging Matrix (TIM), software revision vb17, Siemens, Erlangen, Germany). The modifications had no impact on most pulse sequence elements including radiofrequency excitation, BOLD contrast, repetition time and volume of coverage. For each odd image in the EPI time series, a positive phase encoding increment (a “blip”) was added to the beginning of the train of PE gradient blips. For each even image, a negative blip was added. This resulted in a two-unit shift (Δ_*k*_ = 2) in k-space between odd and even complex images, denoted by *I*_*l*_ and *I*_*m*_, acquired at time points *l* and *m*, where *l* and *m* are odd and even numbers, respectively. The original PLACE implementation [19] used a one-unit shift (i.e., Δ_*k*_ = 1) in k-space between the two complex images; in preliminary development stages, however, it was observed that increasing the shift to two units yielded less noisy “displacement maps”, as subsequently defined below.

In the original PLACE implementation [19], phase ramp differences are expected in the PE direction within image pairs according to the Fourier shift theorem. In the absence of magnetic field inhomogeneity, the effect is described by

$$I_{l}I_{m}^{*}=M_{l}M_{m}\text{exp}\left(i\frac{2\Delta_{k}\pi }{FOV}y\right),\;\;-\frac{FOV}{2}<y<\frac{FOV}{2}$$ (1)

Where *M*_*l*_ and *M*_*m*_ are the magnitude of images *I*_*l*_ and *I*_*m*_, respectively; the spatial coordinate *y* is measured from the isocenter of the PE gradient and represents the physical coordinate in the undistorted images; and *FOV* is the field of view in PE direction. From (Eq 1), *y* can be extracted as

$$y=\frac{FOV}{2\Delta_{k}\pi }Arg\left(I_{l}I_{m}^{*}\right).$$ (2)

When magnetic field inhomogeneity is present, however, the resulting geometric distortion implies that in each image, the signals will be mis-located to position *y*′ instead of their true location *y*. The spatial shift can be determined simply by applying a linear phase ramp along the PE direction to $I_{l}I_{m}^{*}$, creating a new complex image *C*,

$$C=I_{l}I_{m}^{*}\text{exp}\left(-i\frac{2\Delta_{k}\pi }{FOV}y^{\prime }\right)=M_{l}M_{m}\text{exp}\left(i\frac{2\Delta_{k}\pi }{FOV}\left(y-y^{\prime }\right)\right).$$ (3)

It can be anticipated that an operation analogous to (Eq 2) provides a displacement map of the difference, Δ*y* = *y−y*′. (This removes the need to obtain field maps directly, although field maps can be estimated from Δ*y* if needed [28]). However, “crack” and “pile-up” artifacts must be considered, as well as noise. For example, if an image *P* pixels wide is directly stretched into a larger image *Q* pixels wide (*P < Q*), there will be at least *Q−P* empty pixels (cracks) in the output image. Conversely, compressing an image *P* pixels wide into a smaller region *Q* pixels wide (*P > Q*) will result in some pixels receiving signals from more than one source (pile-ups). To address these problems, according to [19], the complex image *C* is expanded in the PE direction by copying each pixel 100 times and performing spatial smoothing. The displacement map Δ*y* is robustly extracted from the expanded and smoothed complex image, *C*_*ES*_, as

$$\Delta y=\frac{FOV}{2\Delta_{k}\pi }Arg\left(C_{ES}\right).$$ (4)

Distorted images *I*_*l*_ and *I*_*m*_ are then expanded in the PE direction in an analogous fashion, corrected pixel-by-pixel according to Δ*y*, then re-binned back to original size.

In the present work, PLACE was performed after EPI data acquisition using custom software developed in MATLAB (the MathWorks, Natick, MA). Two additional steps were included other than the image processing pipeline summarized above. First, additional noise reduction was implemented by complex averaging of data acquired with a multi-channel head coil receiver. Complex averaging not only reduces the overall noise but also leads to a robust combination of multi-channel data by allowing a magnitude weighting which emphasizes the contribution of regions with higher signals from each channel [29]. Mathematically, multi-channel data were thus combined over all *N*_*c*_ channels according to

$$C=\frac{1}{N_{c}}\;\overset{N_{c}}{\underset{n=1}{\sum }}M_{l}\left(n\right)M_{m}\left(n\right)\text{exp}\left(i\frac{2\Delta_{k}\pi }{FOV}\left(y\left(n\right)-y^{\prime }\right)\right).$$ (5)

A second, additional procedure was undertaken to ensure that head motion was negligible within EPI pairs. As the initial processing of human fMRI data involved use of a rigid-body registration algorithm (see below) it was possible to use estimates of head motion as constraints. Registration was run on combined (root-sum-of-squares) magnitude images from the multi-channel head coil. Assuming that geometric distortion was not significantly affected by very small displacements, a difference threshold of 0.05 mm for translations and 0.1° for rotations was set to assign image pairs. The search for suitable image pairings started by comparing the head position for each image to that of its neighbouring image in the time series. If neither of the adjacent time points were below the head motion threshold, the search was expanded in time until the difference-threshold conditions were met. Based on the task-based fMRI design (see below), searches were expanded as necessary from the same block of a given task to adjacent task blocks, mitigating the potential for phase confounds arising from the BOLD effect in task and rest conditions.

To perform static geometric distortion correction (sPLACE), the PLACE displacement map obtained from the first pair of EPI images in the time series was applied to all the EPI images in the fMRI time series. An additional approach was included for dynamic geometric distortion correction (dPLACE), referred to as Displacement Map Averaging (DMA). This approach enabled noise reduction by averaging displacement maps that were obtained for "identical" head positions as determined using the thresholds indicated immediately above. DMA is based on the fact that although ultimately there is a requirement for dynamic geometric distortion correction, the temporal resolution of this correction does not need to be equivalent to that of the fMRI time series data collection. Moreover, the group mean and standard deviation of the number of independent displacement maps that were averaged in DMA was investigated in the human fMRI experiments.

#### Dynamic Off-Resonance in K-space (DORK)

The DORK method assumes that respiration causes a time-dependent frequency shift at a given slice location, leading to linear phase accumulation as EPI data are acquired in k-space. Two phase measurements at different time points are used to estimate both the initial phase of the MRI signal and the frequency offset [27]. This is achieved using a navigator echo (a simple free induction decay without PE) acquired shortly after radiofrequency excitation, and the EPI data acquired at the center of k-space at the echo time *TE*. From these data, acquired at point *t* in the time series, it is possible to estimate the frequency shift Δ*ω*_*t*_ *= ω*_*t*_*−ω*_*r*_ and phase shift Δ*φ*_*t*_ *= φ*_*t*_*−φ*_*r*_ in relation to the reference time point *r*. The EPI k-space data *S*(*t*) are subsequently processed to yield corrected signals *S*′(*t*) according to

$$S\prime \left(t\right)=S\left(t\right)\text{exp}\left(-i\left(\Delta \omega_{t}t+\Delta \varphi_{t}\right)\right).$$ (6)

The vendor-supplied EPI implementation incorporates three optional navigator echoes at the beginning of each slice acquisition to correct for Nyquist ghosts [30]. Data from the second navigator echo were used to enable DORK correction without further modifications to the pulse sequence. (Eq 6) was performed separately for each channel after data acquisition, again using custom MATLAB software. Corrections were applied independently to odd and even images in the time series because of the k-space shift between the two data sets.

### MRI Experiments

All imaging was performed at Baycrest Hospital in Toronto, using a 12-channel "matrix" head coil receiver on the MRI system, and the body coil for radiofrequency transmission. For all imaging sessions, initial tuning included second-order shimming. Multi-slice single-shot EPI (modified to enable dPLACE and DORK) was employed with TE/TR/flip angle = 30 ms/2000 ms/40°, FOV = 204 mm, 32 oblique axial slices 3.5 mm thick, acquisition matrix = 68 × 68, and 0.47 ms echo spacing.

#### Phantom Experiments

Static phantom experiments were designed to show how respiratory effects can corrupt the correction provided when dPLACE is performed alone, and how the combined approach of DORK+dPLACE+DMA can provide static geometric distortion correction in the presence of respiratory effects. The imaging experiments were performed on a 17 cm- diameter spherical agar phantom constructed based on recommendations by the functional Bioinformatics Research Network (fBIRN) Consortium [31] and providing coil loading and MRI characteristics resembling those of the human brain. During the scans, a lung phantom was positioned next to the fBIRN phantom. The lung phantom, subsequently described as "simulated lungs", consisted of two empty plastic bags, each with a volume of approximately 3 L, similar to the ventilated volume of the average human lung. The two bags were covered by light wet towels, and were connected by plastic tubing (2.5 cm diameter) to a breathing mask located outside the magnet bore, 15 cm away from the phantom. The breathing mask was used to inflate and deflate the plastic bags and move the wet towel to introduce dynamic respiration-induced off-resonance effects. Two “runs” (a and b) were performed without use of the breathing mask ("respiration absent"), and an additional two runs (c and d) were performed with a healthy volunteer free-breathing through the mask to generate off-resonance effects for the duration of each scan ("respiration present"). A pneumatic belt (BioPac, Goleta, USA) was placed around the abdomen of the volunteer and the resultant recordings were used as a surrogate measure of ventilation volume in the simulated lungs. Runs b and d were obtained with TR = 80 ms for a single axial slice using the scan parameters that were otherwise identical to runs a and c (as presented above). The high temporal resolution scans were undertaken as an experimental control condition, to evaluate the effect of the simulated lungs on the phase evolution in the EPI time series acquired at lower temporal resolution.

#### Human fMRI Experiments

Six healthy right-handed subjects (2 males and 4 females, average age 27, range 22–32) were imaged. All experiments on human volunteers were performed with the approval of the research ethics board of Baycrest. All subjects gave written informed consent to participate before their imaging session. Foam padding was placed around each the head of each subject to limit motion. Each subject underwent a resting-state and task-based fMRI run. In the resting-state run (e) the subjects were instructed to close their eyes and relax. During the task-based run (f), the subjects performed task-based fMRI involving self-paced bilateral finger tapping tasks with eight alternating 20 s blocks of “task” and “rest” conditions. This run was initiated with a rest block of 28 s, with data discarded from the initial 8 s duration to ensure that magnetization reached the steady state. A summary of all the imaging runs is provided in Table 1.

**Table 1:** Summary of all imaging runs.

Run	Specifications
a	phantom, respiration absent, TR = 2000 ms
b	phantom, respiration absent, TR = 80 ms
c	phantom, respiration present, TR = 2000 ms
d	phantom, respiration present TR = 80 ms
e	human, resting state, TR = 2000 ms
f	human, task-based, TR = 2000 ms

During task-based fMRI, subjects were presented with a cue to start and stop self-paced bilateral finger tapping. Visual stimuli for each block were back-displayed on a projection screen at the rear of the magnet bore using a liquid crystal display projector (Revolution III, Boxlight 6000, Boxlight Corp, Belfair, WA) through a waveguide in the radiofrequency shield, and viewed by subjects using angled mirrors attached to the top of the head coil. The task was programmed in E-Prime (Psychology Software Tools Inc, Sharpsburg, PA) and lasted 348 s. Subjects were instructed to breathe normally while remaining as still as possible during imaging. The respiratory cycles were monitored during the fMRI using the pneumatic belt, as described above. In addition, all subjects were initially imaged with a T1-weighted 3D magnetization-prepared rapid gradient-echo sequence (TE/ flip angle = 2.63ms/6, 1500 ms between inversion preparation pulses, FOV = 256 mm, 160 slices 1 mm thick, and acquisition matrix = 256 × 256), providing images to serve as anatomical reference.

### Post-processing Methods

For both phantom and human fMRI experiments, all complex k-space data from each channel were initially reconstructed using custom MATLAB software, with the minimal "baseline" processing consisting of regridding, apodization, and Nyquist ghost correction [30]. To evaluate the performance of sPLACE and dPLACE (with and without DMA and DORK) six different incremental post-processing strategies were investigated: 1) baseline processing to yield magnitude images; 2) sPLACE, 3) dPLACE, 4) dPLACE with DMA, 5) DORK followed by dPLACE, and 6) DORK followed by dPLACE with DMA. Datasets a, c, e and f were processed according to the strategies 1–3 and 6. The remaining strategies, 4 and 5 were only applied to the phantom datasets (a and c) to add additional insight into the comprehensive post-processing strategy (i.e. strategy 6). For ease of presentation, the various acquisition-post-processing combinations are subsequently referred to by a letter-number pair. For example, a2 represents the phantom experiment with respiration absent, EPI with TR = 2000 ms, and post-processing with sPLACE correction.

The phase images of datasets b and d were spatially unwrapped using the PRELUDE algorithm [32] of the FMRIB Software Library (FSL; http://www.fmrib.ox.ac.uk/fsl), followed by temporal unwrapping and detrending using MATLAB. Again, the temporal unwrapping was performed independently for odd and even images in the time series. The correlation between the recorded ventilation volume and the time evolution of the spatially-averaged phase images was calculated in MATLAB for odd and even images in the time series individually to assess the performance of the simulated lungs in producing magnetic field fluctuations. A temporal shift was applied to the phase time series to maximize its correlation with the ventilation volume time-course.

Retrospective motion correction was performed using the rigid-body volume registration function in Analysis of Functional NeuroImages (AFNI) software package [33] by aligning all the images in the times series to the tenth image. Moreover, the peak-to-peak (p-p) values were obtained for all six motion parameter estimates (three translation and three rotations) for each subject and the group statistics (mean, standard deviation and range) of the p-p values for each motion parameter were calculated. The directions with the largest p-p range of rotation and translation were determined.

To quantify image stability across fMRI time-series data, temporal standard deviation (tSD) maps were generated using MATLAB for phantom experiments (a and c) and for human imaging with subjects at rest (e). The tSD value was calculated for each voxel after detrending and motion correction, and was reported as a percentage by normalizing with respect to the mean signal amplitude over the time series. For human imaging, a binary brain mask containing only grey matter and white matter was extracted from the anatomical scans aligned to the motion-corrected fMRI data using SPM12 (Welcome Trust, London, UK). The mask was then subsequently applied to all points in the image time series so that only voxels containing brain tissue were included in the analysis. The spatially averaged temporal standard deviation, $\bar{\text{tSD}}$, was also calculated over the whole brain volume as well as over grey matter (the primary source of BOLD signals). The $\bar{\text{tSD}}$ values for sPLACE (e2), DORK+dPLACE+DMA (e6) and the baseline (e1) were compared for statistical significance using two separate directional one-tailed paired t-tests over all six subjects to test the hypothesis that $\bar{\text{tSD}}$ is larger with either e1 or e2 post-processing compared to use of e6. Datasets e3 and f3 were not processed further.

For task-based fMRI, the dPLACE displacement maps after DORK and DMA were assessed by calculating the maximum displacement during the time interval of the fMRI experiment for each voxel and then averaging over the brain volume, yielding the spatially averaged maximum displacement. This metric provides insight about the magnitude of the motion-induced dynamic geometric distortion present in the datasets. Additionally, activation maps were obtained using AFNI after performing slice timing correction; volume registration; detrending; spatial smoothing (Gaussian kernel with 4 mm full width at half maximum); temporal smoothing (3 point median filter); censoring volumes with rotations or displacements > 0.3° or mm, respectively; masking fMRI signals to zero outside the brain; and general linear model (GLM) analysis [34]. The GLM was conducted using a “task waveform” boxcar function (with unity amplitude during task blocks and zero during rest blocks) convolved with the canonical BOLD hemodynamic response waveform available in AFNI. Third order polynomial coefficients and the six head motion parameters were also included as nuisance regressors. The results of the GLM analysis were summarized by color activation maps with the threshold for statistical significance determined by correcting for multiple comparisons according to the false discovery rate (FDR) of q = 0.01[35]. Activation maps were then overlaid on the respective anatomical images of each subject (previously aligned to the fMRI data) using affine registration in AFNI.

The quality of activation maps was assessed by qualitative visual inspection as well as by two quantitative approaches. First, the number of active voxels (counted over the whole brain) was compared for DORK and dPLACE with DMA (f6), the baseline (f1), and the sPLACE (f2) post-processing to test the hypothesis that voxel counts increase with use of f6 compared to f1 and f2. Second, the difference between the fitted curve obtained by GLM analysis and the time series was calculated for f6, f1 and f2, yielding voxel-wise residual error signals for each subject. The voxel-wise temporal standard deviation of each residual error signal was then calculated, averaged over the brain volume for each subject, and submitted to two one-tailed paired t-tests as described above. The analogous calculations were also performed for the temporal standard deviation of the residual error signal averaged over grey matter.

## Results

### Phantom Experiments

The time evolution of the spatially-averaged phase images (from all channels of the coil combined by complex averaging) was compared with the associated respiratory signal recorded in both the absence (run b) and presence (run d) of respiration. Cross-correlation coefficient values of –0.05±0.02 (–0.05±0.02) and –0.74±0.23 (–0.75±0.25) were found for odd (even) images in the time series data for runs b and d, respectively. These results indicated that the simulated lungs induced off-resonance field fluctuations that were characteristic of respiration.

Because the phantom was stationary during the experiment, the nearest neighbour (adjacent time point) pairing for dPLACE correction always satisfied the displacement threshold. To investigate dPLACE with DMA, the number of averages was limited to 8 to match the practical value observed in human subjects at rest (see human fMRI experiments below).

**Fig 1:**
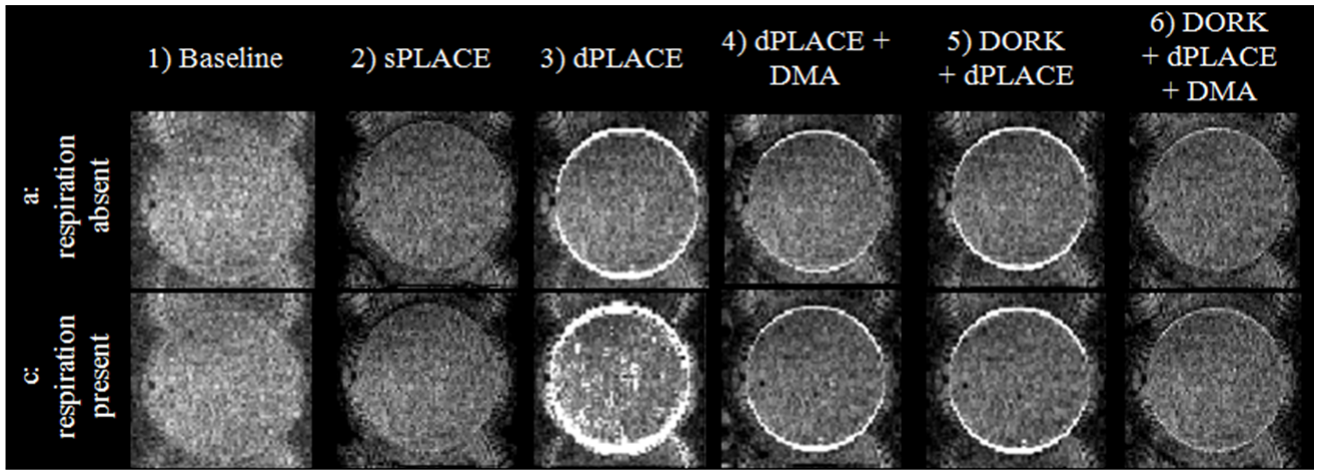
Phantom experiment results: Temporal standard deviation (tSD) maps in the absence (first row) and presence (second row) of simulated respiration after: 1) no further processing, 2) sPLACE, 3) dPLACE, 4) dPLACE with DMA, 5) DORK and dPLACE, and 6) DORK and dPLACE with DMA.

Fig 1 illustrates the effect of the six post-processing stages (1–6) on tSD values in the absence (run a) and presence (run c) of respiration. First comparing tSD values for a1 and c1, very little difference is observed, indicating that the off-resonance fluctuation caused by the simulated lungs had little effect on magnitude images. This is expected, as the fluctuations in the phase images are known to be much more pronounced [36]. In both a1 and c1, tSD values are appreciable both in regions of Nyquist ghosting and outside the extent of the phantom. There is a very slight trend towards increased tSD values at the upper and lower edges of the phantom in c1 in comparison to a1, which could be indicative of off-resonance effects from simulated respiration. However, since the phantom experiment was conducted with a static phantom, the geometric distortions did not change significantly in a1 and c1 baseline images (i.e., no observable edge artifacts). The application of sPLACE (a2 and c2) has significantly reduced the tSD compared to a1 and c1 respectively, with marginally lower values in absence of respiration (a2) compared to in presence of respiration (c2). This indicates that sPLACE has effectively reduced the static geometric distortion irrespective of the off-resonance frequency fluctuations induced by respiration.

Ideally, in the absence of motion, dynamic geometric distortion correction is expected to perform equivalently to static geometric distortion. Correction using dPLACE alone, however, increases tSD markedly when off-resonance effects from respiration are present (c3). The tSD enhancement observed at the upper and lower edges of the phantom (in the PE direction) suggests that the dPLACE displacement maps contain noise at these locations. The additional noise is likely due to off-resonance respiratory-induced fluctuations as well as partial volume effects that are emphasized as a result of spatial expansion and heavy smoothing involved in dPLACE correction. For a3 in relation to a1, tSD has slightly reduced inside the phantom, and substantial reductions are observed outside the phantom in regions of residual Nyquist ghosts. The true extent of the phantom is better visualized, indicating effective geometric distortion correction, with some elevation in tSD at the edges of the phantom in the y direction due to the partial volume effects discussed above. This difference between a3 and c3 is expected as dPLACE suppresses geometric distortion but is sensitive to off-resonance frequency fluctuations between image pairs, as previously shown [26][25].

An improvement is achieved by performing dPLACE with DMA, and is clearly evident both in the absence and presence of respiration (a4 and c4, respectively). Use of DMA not only suppresses the cyclic off-resonance fluctuations through averaging, but also reduces the random noise present in the PLACE displacement maps. However, small but noticeable tSD elevations are still observed at the edges of the phantom in a4 and c4,. Other than these rim effects, tSD values in a4 and c4 are remarkably similar. The DORK+dPLACE post-processing (a5 and c5) produces tSD values that are substantially improved over the application of dPLACE alone (a3 and c3), and with values within the phantom that are very similar irrespective of whether respiration was present or absent, very similar to dPLACE+DMA results (a4 and c4). However, DORK+dPLACE post-processing shows slightly elevated tSD at the rim of the phantom in comparison to dPLACE+DMA, likely due to experimental error in estimating off-resonance frequency using DORK. Lastly, the final column of Fig 1 shows the results of DORK+dPLACE+DMA (a6 and c6), which demonstrate the best tSD suppression over all of pipelines 3–6: low tSD values within the phantom as well as at the edges due to the use of temporal averaging. No substantial differences in the tSD values for c6 and a6 are observed, indicating that robust static geometric distortion is achieved with correction of off-resonance effects from simulated respiration. Also, comparing DORK+dPLACE+DMA (a6, c6) with sPLACE (a2, c2) no visible difference is observed except for very small increases at the edge of the phantom in a6 and c6. Overall, Fig 1 shows improved temporal standard deviation as a result of using DORK+dPLACE+DMA and good correction for static geometric distortion in presence of respiratory effects. Moreover, the phantom experiments demonstrate how respiratory effects can corrupt the correction provided when dPLACE is performed alone, and how the combined approach of DORK+dPLACE+DMA can provide static geometric distortion correction in the presence of respiratory effects that matches what can be achieved with sPLACE.

### Human fMRI Experiments

**Fig 2:**
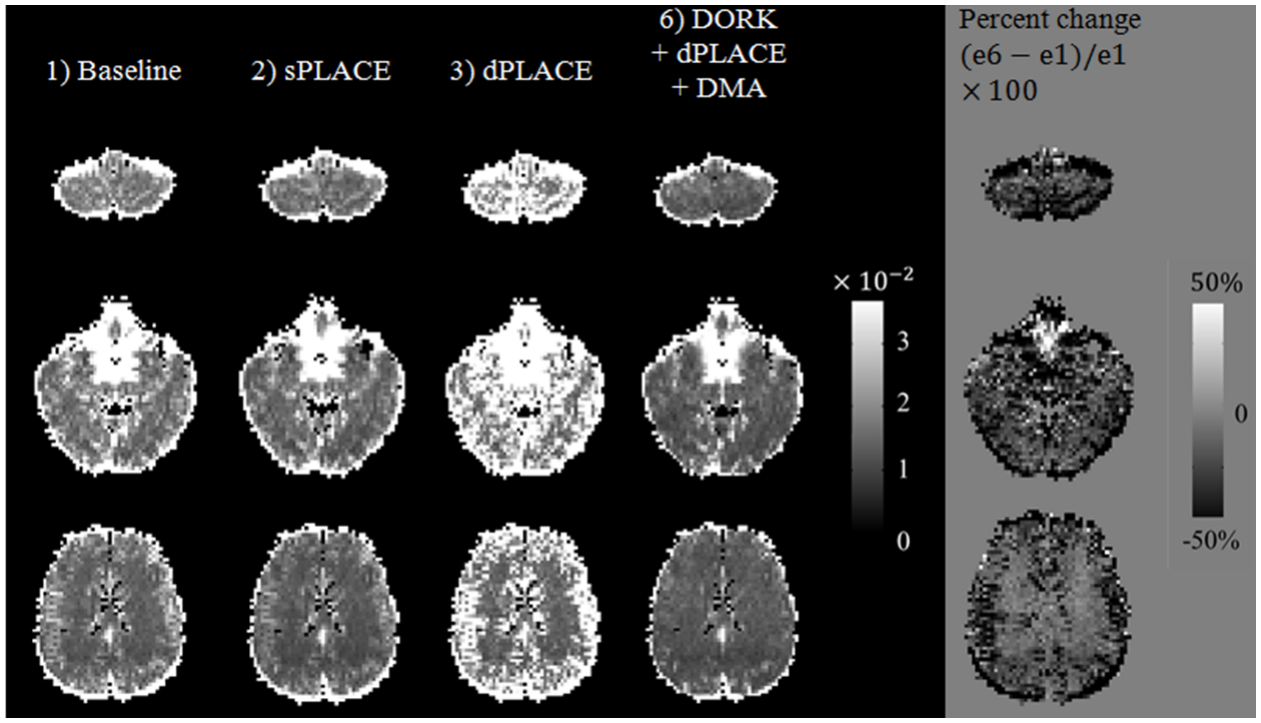
Group mean, standard deviation, and range of p-p head motion for time series data collection for subjects (a) at rest and (b) during task-based fMRI. ΔSI, ΔRL, and ΔAP denote displacements in the superior-inferior, right-left, and anterior-posterior directions, respectively; roll, pitch and yaw denote the angular rotations about the SI, RL and AP axes, respectively.

Although the human fMRI experiments were conducted in young healthy adults and using foam head restraints, small but non-trivial head motion estimates were found through motion correction in AFNI. The group mean, standard deviation, and the range of the p-p angular rotations (roll, pitch, yaw) and displacements (ΔSI, superior-inferior; ΔRL, right-left; and ΔAP, anterior-posterior) are shown in Fig 2a and 2b for subjects at rest and during task-based fMRI, respectively. At rest, the group mean p-p values (black bars) were ≤0.7° over all angular rotations, and ≤0.7 mm over all displacement axes; with group standard deviations (white bars) of ≤0.2° and <0.3 mm, respectively; and ranges (error bars) of ≤1.3° and ≤1.7 mm, respectively. During task-based fMRI, head motion was slightly elevated with analogous group mean p-p values of ≤0.9° and ≤1 mm, group standard deviations of ≤0.3° and ≤0.4 mm, and ranges of ≤2.5° and ≤1.9 mm, respectively. For both at-rest and task-based runs, the largest extents of motion were observed in ΔAP and pitch rotation parameters (p<0.05 as measured by Tukey range tests comparing ΔAP with ΔRL and ΔAP, and comparing pitch values with roll and yaw values). Overall, however, motion was constrained to a fraction of the voxel dimension, with even smaller changes observed from time point to time point. Nearest neighbor pairing (ie., sequential images in the fMRI time series) was achieved for dPLACE correction for 90% of points with subjects at rest, and 87% of points during task-based fMRI. For the 13% of points remaining in the latter case, 9% achieved pairings within the same task block, and 4% achieved pairings within adjacent blocks in the fMRI time series.

For dPLACE with DMA, the number of averaged displacement maps had means of 6.3 and 8.5, and standard deviations of 3.8 and 4.6, respectively, for the tasked-based fMRI and for imaging with subjects at rest, over all subjects and time points.

**Fig 3:**
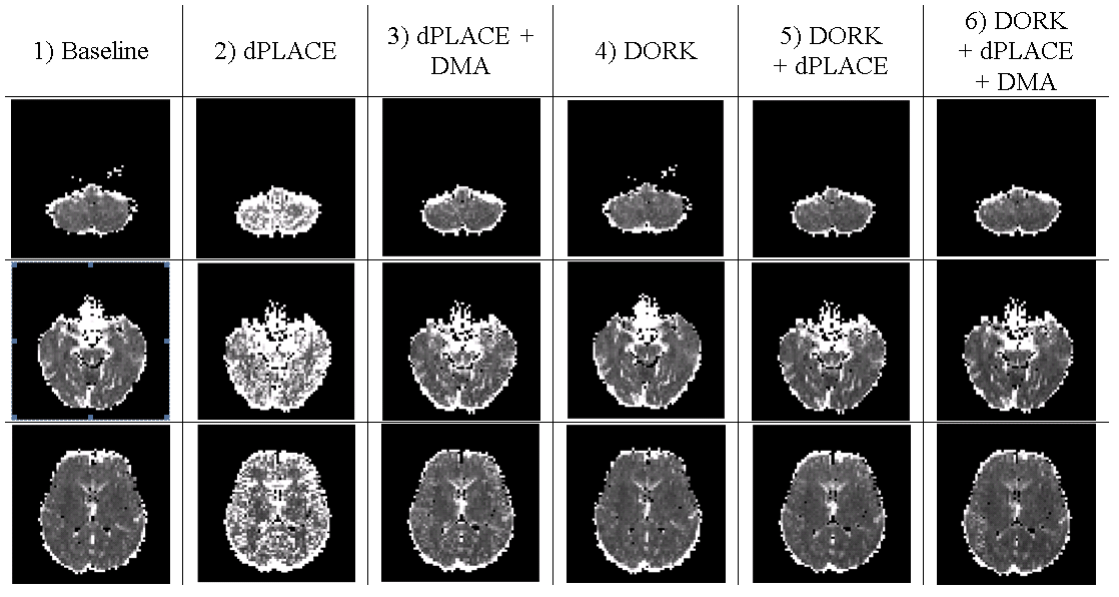
tSD maps of three different slices for at rest scans (scan e) of a representative subject after: 1) no further processing, 2) sPLACE, 3) dPLACE, and 6) DORK and dPLACE with DMA. Maps of the percent signal change achieved by the combined approach 6) compared to no further processing 1) are shown at the far right.

Fig 3 shows tSD values for three slice locations from a representative subject at rest (run e) corresponding to the six post-processing stages. The investigation of several slices is important, as previous literature indicates that off-resonance effects are more pronounced in slices that are more inferior and closer to the lungs [37]. In relation to baseline processing (e1), tSD values after dPLACE correction were noticeably increased due to respiratory-induced off-resonance effects especially in the inferior slice location (e3). However, after sPLACE correction (e2) tSD values were visibly decreased but with small edge artifacts still present. The use of DORK+dPLACE+DMA (e6) provided effective dynamic geometric distortion correction and improved tSD values over the baseline (e1) as well as sPLACE (e2), as also indicated by maps of percent signal change shown in Fig 3 (far right column). The abovementioned trends were observed in all subjects, with a significant reduction in whole-brain $\bar{\text{tSD}}$ value for DORK+dPLACE+DMA compared to use of baseline post-processing (p < 0.05). In addition, there was a trend toward reduced whole-brain $\bar{\text{tSD}}$ values for DORK+dPLACE+DMA compared to use of sPLACE (p = 0.26) that approached significance when only the grey matter $\bar{\text{tSD}}$ was considered (p = 0.09).

**Fig 4:**
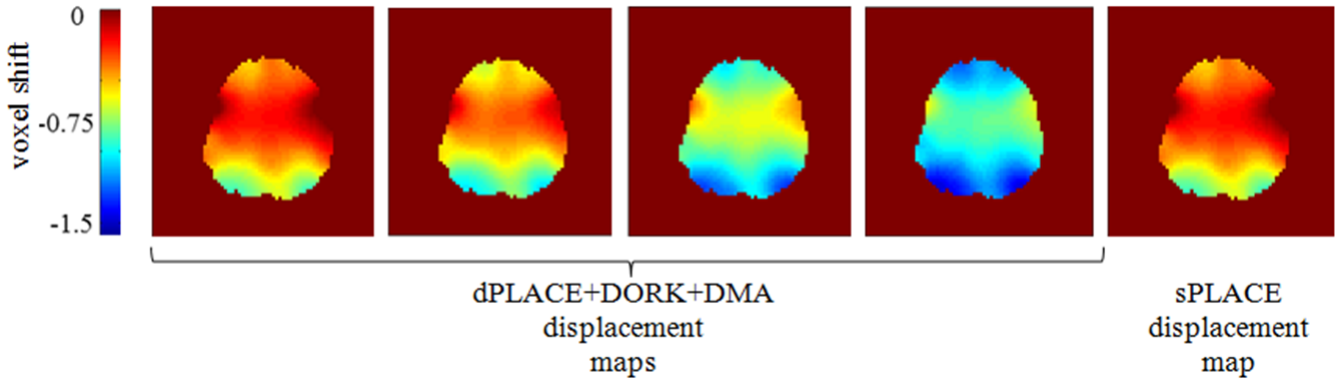
Four sample dPLACE+DORK+DMA displacement maps for a representative subject at different time points during task-based fMRI (scan f). The static displacement map generated by sPLACE is shown for comparison.

Fig 4 shows four dPLACE+DORK+DMA displacement maps for task-based fMRI (run f) of the same subject at different points during the image time series, and the associated sPLACE displacement map for comparison. The dynamic displacement maps change visibly over the duration of the fMRI experiment, indicating that the motion-induced dynamic variations in geometric distortion are substantial. More quantitatively, the maximum change in voxel displacement shown in Fig 4 is 1.1 voxels (i.e. 3.3 mm), with a whole-brain average of 0.8 voxels (2.4 mm). The analogous group mean values are 0.7 voxels (2.1 mm) and 0.5 voxels (1.5 mm) for task-based fMRI and resting fMRI runs, respectively.

**Fig 5:**
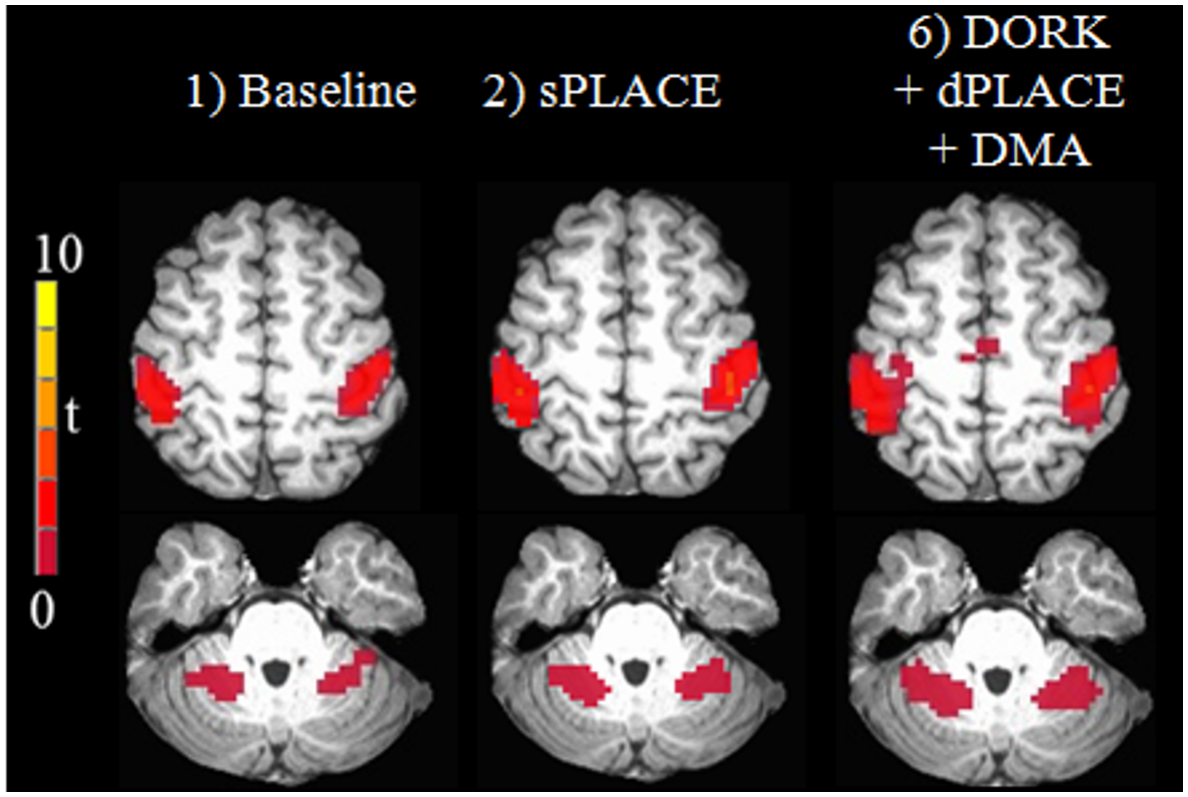
Activation brain maps for a representative subject after no further processing (baseline), sPLACE and DORK+dPLACE+DMA on two brain slices.

**Fig 6:**
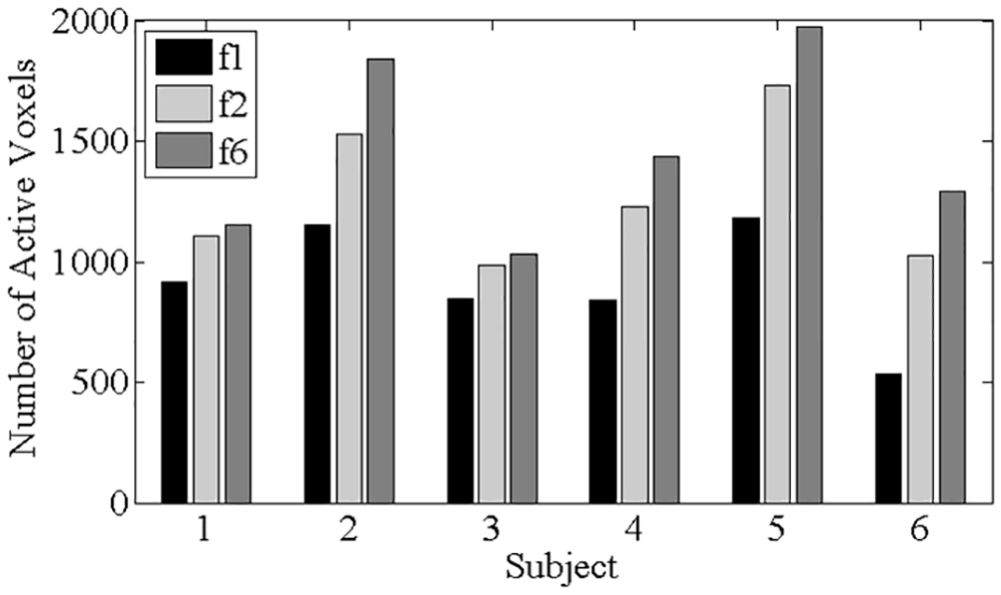
The number of active voxels over the whole brain after baseline (f1), sPLACE (f2), and DORK+dPLACE+DMA (f6) post-processing for all subjects.

Fig 5 shows brain activation maps derived from task-based fMRI (run f) after DORK+dPLACE+ DMA (f6), sPLACE (f2), and baseline processing (f1) for two slices from the same representative subject. Use of sPLACE and DORK+dPLACE+DMA led to an enlargement of the activation areas observed with baseline processing (bilateral primary sensorimotor cortex, cerebellum). In addition, use of DORK+dPLACE+DMA revealed additional activation of the supplementary motor area that was not observed with the other processing strategies. More quantitatively, Fig 6 shows the number of active voxels over the whole brain across subjects, for post-processing according to f6, f2, and f1. Comparing the spatially averaged temporal standard deviation of the residual error signal, whole-brain values for DORK+dPLACE+DMA were significantly reduced compared to those obtained with baseline post-processing (p < 0.05); and showed a trend toward reduction when compared to use of sPLACE (p = 0.18). In addition, the temporal standard deviation of the residual error signal showed a statistically significant reduction for DORK+dPLACE+DMA compared to use of sPLACE, when spatial averaging was conducted over grey matter only (p < 0.05).

## Discussion

This study demonstrates how respiratory effects compromise the PLACE approach for dynamic correction of geometric distortion during fMRI. Robust dynamic correction is achievable by combining DORK and displacement map averaging (DMA) methods prior to executing dPLACE for typical whole-brain fMRI datasets with repetition times of ~2–3 s. The ramifications of the work are discussed below.

First, static phantom experiments with and without cyclic off-resonance fluctuations typical of respiration were performed to implement, debug, and provide early validation of dPLACE. During this process, it was observed that shifting the EPI raster by Δ_*k*_ = 2 yielded less noisy displacement maps compared to shifting by Δ_*k*_ = 1, particularly in the regions of residual Nyquist ghost. This benefit likely arose because the use of Δ_*k*_ = 2 resulted in the same direction of k-space line traversal between each set of two images paired together for PLACE correction, helping to reduce errors in the echo alignments of k-space data that occur with use of Δ_*k*_ = 1. The optimal choice of Δ_*k*_ is likely to be scanner-dependent, and is beyond the scope of the present work.

The phantom experiments were also designed as a preliminary step to investigate the impact of various post-processing pipelines for dynamic geometric distortion on image instability, before proceeding to human fMRI experiments. The phantom experiments were designed without bulk motion with the expectation that the selected dynamic geometric distortion approach should first be able to match the ability of sPLACE to correct for static geometric distortion in presence of respiration induced off-resonance effects. To validate the performance of the simulated lungs in inducing magnetic field fluctuations, additional scans with TR = 80 ms were acquired and cross-correlation of the recorded respiratory signal with the global phase fluctuations was calculated both in presence and absence of respiration. The cross-correlation observed between the two time series was within experimental error of the 95% correlation reported in [38] for TR = 250 ms, indicating that the phantom experiment was effective in producing off-resonance field fluctuations characteristic of respiration.

It was hypothesized that DORK combined with dPLACE and DMA would improve image stability in phantoms, and improve statistical inference of brain activity in fMRI of young healthy adults. The results that were obtained in phantoms (Fig 1) were strongly supportive. Irrespective of whether simulated respiration was present or absent, DORK+dPLACE+DMA processing robustly improved tSD maps beyond what was observed in the original data (baseline processing) and matching what was achieved by sPLACE for the static phantom. Improvements were observed inside the phantom, and also outside the phantom in zones of Nyquist ghosts. Use of dPLACE in the absence of DORK and DMA caused substantially elevated tSD values in the presence of simulated respiration due to the impact of off-resonance effects on phase differences between EPI pairs [25][26]. This occurred despite the fact that PLACE includes a spatial smoothing process to prevent cracks and pile ups.

Although dPLACE+DMA and DORK+dPLACE processing produced good results compared to use of dPLACE alone, elevated tSD values were observed at the rim of the phantom irrespective of whether simulated respiration was present or absent. For dPLACE+DMA, the elevations were likely due to the effects of averaging substantial phase changes over a limited number of images, whereas for DORK+dPLACE the experimental error in estimating off-resonance frequency using DORK was the likely cause. However, the DORK+dPLACE+DMA approach provides the desired stability, effectively suppressing the rim effect on tSD values. DMA effectively reduces the residual noise remaining in the DORK-corrected PLACE displacement maps. Note that this approach is valid as long as the PLACE displacement maps that are averaged together are acquired at highly similar head positions, and thus affected similarly by geometric distortion.

In the present work, performing DMA over 8 images in the phantom experiments was sufficient to ensure excellent performance of DORK and dPLACE. This observation also reinforces that although ultimately there is a requirement for dynamic geometric distortion correction, the temporal resolution of this correction does not need to be equivalent to that of the fMRI time series data collection. In other words, the dynamic geometric distortion can be implemented as multiple static geometric distortions during the time interval of fMRI experiment.

The excellent results obtained in phantoms made a strong case for progressing to additional validation and hypothesis testing in humans. In human experiments, even compliant young healthy subjects (such as those studied) exhibit small amounts of head motion that potentially can cause dynamic geometric distortion during fMRI. The strategy for pairing EPI images to undertake dPLACE was developed carefully, therefore, based on stringent threshold criteria for assessing whether substantial head motion had occurred. Adjacent image pairs were found to be acceptable for dPLACE correction in the large majority (~90%) of cases throughout the fMRI time series. For the limited cases in which nearest-neighbor pairing was not acceptable, adequate pairing was achieved by searching within the same block and in very few cases the similar adjacent blocks during task-based fMRI. Similar observations were made in a previous study involving dPLACE (but without DORK and DMA) based on motion information obtained from optical position tracking for real-time head motion correction [39]. This previous study used a more generous displacement threshold and thus for almost all (~98%) images in the time series, adjacent image pairs were judged to be acceptable. The present study and [39] do indicate that the image pairings required for dPLACE are not a substantial limitation of the method in the context of fMRI, for experiments involving young healthy adults.

It was important in the present work to evaluate the temporal resolution for dynamic geometric distortion that was practically achievable to provide optimal dPLACE+DORK processing. Using the same motion threshold for establishing image pairs, on average 6.3±3.8 and 8.5±4.6 displacement maps were averaged for tasked-based and resting fMRI runs over all the subjects and time points. Utilizing the combined DORK+dPLACE+DMA approach for patient populations (that often exhibit larger head motions) is expected to reduce the number of averaged PLACE displacement maps and may reduce the effectiveness of DORK+PLACE+DMA for fMRI applications. It is possible that this issue can be mitigated by increasing the displacement threshold slightly while maintaining adequate correction for geometric distortions. Optimizing the displacement threshold is beyond the scope of the present work, however.

Although small amounts of head motion were present in the human fMRI data, dynamic geometric distortion correction (DORK+dPLACE+DMA) proved to be beneficial. Statistically significant improvements were observed in whole-brain tSD (in the resting run) and the number of active voxels and temporal standard deviation of residual error (in the task-based run), relative to baseline processing. Similar trends were observed when comparing DORK+dPLACE+DMA results to those obtained with sPLACE, which achieved statistical significance specifically for task-based fMRI runs when the temporal standard deviation of the residual error was spatially averaged over grey matter. Observing the DORK+dPLACE+DMA displacement maps at various points in the image time series was a useful part of interpreting the results given immediately above. The small head motions that were observed resulted in dynamic changes in geometric distortion that were often a substantial fraction of the voxel size. This suggests that the corrections provided by DORK+dPLACE+DMA would be useful in widespread fMRI applications involving young healthy adults.

It has already been mentioned that if the DORK+dPLACE+DMA approach is to be used in the presence of more substantial head motion, further investigations will be required to optimize the DMA procedure. One issue is the number of images that must be averaged together; another is the type of head motion exhibited by the subject. For example, slowly varying head motions are likely more tractable than more rapid or high-frequency motions. It would be interesting to investigate the robustness of DMA in this regard, and more generally, the PLACE+DORK+DMA approach in populations known to exhibit more problematic head motion (such as stroke patients). However, this is substantially beyond the scope of the present paper as such work would need to include some version of real-time motion correction into the fMRI protocol to satisfy the requirement that PLACE is implemented on EPI pairs that are of the same slice plane [40–43].

As expected, some subjects showed more substantial benefit from the DORK+dPLACE+DMA pipeline than others, likely because of subject-dependent anatomical and physiological variations in the amplitude and spatial pattern of respiration-induced off-resonance field fluctuations [37]. Depending on the subject, there may be residual off-resonance effects even after DORK correction, as the DORK method assumes that respiration causes a global frequency shift within a given image slice. More sophisticated approaches incorporate a spatial distribution of frequency shifts [37], which may be pertinent in the inferior slices closer to the lung cavity, and at higher static magnetic fields. However, the present results indicate that the need to account for a spatial distribution is relatively minor for axial images acquired at 3 T.

It should also be noted that the DORK correction may not perform as well for sagittal and coronal images, where off-resonance effects are more spatially variable within a given slice. In such cases, other off-resonance correction techniques may be employed effectively to make dPLACE practical [44]. For example [45], the authors have recently compared the performance of several physiological noise correction techniques for mitigating the effects of respiration on PLACE displacement maps, including nuisance variable regression (NVRk) [46], retrospective image-based correction (RETROICOR) [44] and DORK. It was found that among the tested techniques, DORK showed the most promise. Moreover, cardiac effects are also an important noise source in fMRI data (especially those acquired in the resting state). Correction of cardiac noise is not provided by DORK+dPLACE+DMA, as this noise occurs at a higher frequency than both respiratory and head motion, with a complex dependence that relates to the spatial organization of the cerebrovasculature. Nevertheless, the fMRI data acquired and processed by the PLACE+DORK+DMA approach remains amenable to concurrent use of other signal processing approaches to remove cardiac noise (such as RETROICOR).

Considering the fMRI activation maps further, it should be noted that the “ground truth” (i.e., precise knowledge of the true spatial pattern of brain activity associated with the self-paced bilateral finger tapping tasks) is unavailable. In addition, an increase in the number of activated voxels by itself is not necessarily an indication of performance improvement. Similar to the temporal standard deviation at rest, residual errors in the GLM analysis of the task-based fMRI data were also investigated. A statistically significant improvement was observed across the group of subjects as a result of the DORK+dPLACE+DMA approach, compared with baseline processing.

Overall, this study provides a proof-of-concept for practical application of PLACE to correct for dynamic geometric distortion in fMRI time series. Respiratory-induced off-resonance field fluctuations were demonstrated to have an adverse effect on use of dPLACE for human fMRI with typical long repetition times. Correcting for global off-resonance frequency fluctuations using DORK and DMA enabled application of dPLACE with reduced temporal standard deviation and improved activation map quality. With the aim of developing a robust and comprehensive solution for head motion correction, combining dynamic geometric distortion using PLACE with a real-time motion correction technique is a potential area of future interest, including the application of such work to challenging patient populations.

## Abbreviations

BOLD: blood oxygenation level dependent
DMA: displacement map averaging
DORK: dynamic off resonance in k-space
dPLACE: dynamic phase labeling for additional coordinate encoding
EPI: echo planar imaging
fMRI: functional magnetic resonance imaging
PE: phase encoding
PLACE: phase labeling for additional coordinate encoding
sPLACE: static phase labeling for additional coordinate encoding

## References

[1] Bandettini P a., Wong EC, Hinks RS, Tikofsky RS, Hyde JS. Time course EPI of human brain function during task activation. Magn Reson Med [Internet]. 1992 6;25(2):390–7. Available from: http://doi.wiley.com/10.1002/mrm.1910250220 10.1002/mrm.1910250220 1614324

[2] Kwongt KK, Belliveaut JW, Cheslert DA, Goldbergt IE, Weisskofft RM, Poncelett BP, et al Dynamic magnetic resonance imaging of human brain activity during primary sensory stimulation. 1992;89(6):5675–9. 10.1073/pnas.89.12.5675 PMC49355 1608978

[3] Ogawa S, Tank D, Memon R, Ellerman J, Kim S-G, Merkle H, et al Intrinsic signal changes accompanying sensory stimulation: Functional brain mapping with magnetic resonance imaging. 1992;89(7):5951–5. 10.1073/pnas.89.13.5951 PMC402116 1631079

[4] Mansfield P. Multi-planar image formation using. 1977;10:55–8.

[5] Jezzard P, Balaban RS. Correction for geometric distortion in echo planar images from B0 field variations. Magn Reson Med. 1995;34(1):65–73. 7674900 10.1002/mrm.1910340111

[6] Reber PJ, Wong EC, Buxton RB, Frank LR. Correction of off resonance-related distortion in echo-planar imaging using EPI-based field maps. Magn Reson Med [Internet]. 1998 2;39(2):328–30. Available from: http://www.ncbi.nlm.nih.gov/pubmed/9469719 10.1002/mrm.1910390223 9469719

[7] Ojemann JG, Akbudak E, Snyder a Z, McKinstry RC, Raichle ME, Conturo TE. Anatomic localization and quantitative analysis of gradient refocused echo-planar fMRI susceptibility artifacts. Neuroimage [Internet]. 1997 10;6(3):156–67. Available from: http://www.ncbi.nlm.nih.gov/pubmed/9344820 10.1006/nimg.1997.0289 9344820

[8] Devlin JT, Russell RP, Davis MH, Price CJ, Wilson J, Moss HE, et al Susceptibility-induced loss of signal: comparing PET and fMRI on a semantic task. Neuroimage [Internet]. 2000 6 [cited 2015 Feb 2];11(6 Pt 1):589–600. Available from: http://www.ncbi.nlm.nih.gov/pubmed/10860788 10.1006/nimg.2000.0595 10860788

[9] Gorno-Tempini ML, Hutton C, Josephs O, Deichmann R, Price C, Turner R. Echo time dependence of BOLD contrast and susceptibility artifacts. Neuroimage [Internet]. 2002 1 [cited 2015 Feb 2];15(1):136–42. Available from: http://www.ncbi.nlm.nih.gov/pubmed/11771981 10.1006/nimg.2001.0967 11771981

[10] Chen NK, Wyrwicz a M. Correction for EPI distortions using multi-echo gradient-echo imaging. Magn Reson Med [Internet]. 1999 6;41(6):1206–13. Available from: http://www.ncbi.nlm.nih.gov/pubmed/10371453 10.1002/(sici)1522-2594(199906)41:6<1206::aid-mrm17>3.0.co;2-l 10371453

[11] Robson MD, Gore JC, Constable RT. Measurement of the Point Spread Function in MRI Using Constant Time Imaging. (12). 10.1002/mrm.1910380509 9358447

[12] Zaitsev M, Hennig J, Speck O. Point spread function mapping with parallel imaging techniques and high acceleration factors: fast, robust, and flexible method for echo-planar imaging distortion correction. Magn Reson Med [Internet]. 2004 11 [cited 2015 Mar 4];52(5):1156–66. Available from: http://www.ncbi.nlm.nih.gov/pubmed/15508146 10.1002/mrm.20261 15508146

[13] Andersson JLR, Skare S, Ashburner J. How to correct susceptibility distortions in spin-echo echo-planar images: application to diffusion tensor imaging. Neuroimage [Internet]. 2003 10 [cited 2015 Feb 11];20(2):870–88. Available from: http://www.ncbi.nlm.nih.gov/pubmed/14568458 10.1016/S1053-8119(03)00336-7 14568458

[14] Hutton C, Bork A, Josephs O, Deichmann R, Ashburner J, Turner R. Image distortion correction in fMRI: A quantitative evaluation. Neuroimage [Internet]. 2002 5 [cited 2014 Jan 23];16(1):217–40. Available from: http://www.ncbi.nlm.nih.gov/pubmed/11969330 10.1006/nimg.2001.1054 11969330

[15] Andersson JL, Hutton C, Ashburner J, Turner R, Friston K. Modeling geometric deformations in EPI time series. Neuroimage [Internet]. 2001 5 [cited 2014 Oct 8];13(5):903–19. Available from: http://www.ncbi.nlm.nih.gov/pubmed/11304086 10.1006/nimg.2001.0746 11304086

[16] Boegle R, Maclaren J, Zaitsev M. Combining prospective motion correction and distortion correction for EPI: towards a comprehensive correction of motion and susceptibility-induced artifacts. MAGMA [Internet]. 2010 9 [cited 2015 Jan 13];23(4):263–73. Available from: http://www.ncbi.nlm.nih.gov/pubmed/20694501 10.1007/s10334-010-0225-8 20694501

[17] Ooi MB, Muraskin J, Zou X, Thomas WJ, Krueger S, Aksoy M, et al Combined prospective and retrospective correction to reduce motion-induced image misalignment and geometric distortions in EPI. Magn Reson Med [Internet]. 2013 3 1 [cited 2013 Oct 25];69(3):803–11. Available from: http://www.ncbi.nlm.nih.gov/pubmed/22499027 10.1002/mrm.24285 PMC3402592 22499027

[18] Sutton BP, Noll DC, Fessler J a. Dynamic field map estimation using a spiral-in/spiral-out acquisition. Magn Reson Med [Internet]. 2004 6 [cited 2015 Mar 30];51(6):1194–204. Available from: http://www.ncbi.nlm.nih.gov/pubmed/15170840 10.1002/mrm.20079 15170840

[19] Xiang Q-S, Ye FQ. Correction for geometric distortion and N/2 ghosting in EPI by phase labeling for additional coordinate encoding (PLACE). Magn Reson Med [Internet]. 2007 4 [cited 2013 Oct 28];57(4):731–41. Available from: http://www.ncbi.nlm.nih.gov/pubmed/17390358 10.1002/mrm.21187 17390358

[20] Hajnal J V., Myers R, Oatridge A, Schwieso JE, Young IR, Bydder GM. Artifacts due to stimulus correlated motion in functional imaging of the brain. Magn Reson Med [Internet]. 1994 3;31(3):283–91. Available from: http://doi.wiley.com/10.1002/mrm.1910310307 10.1002/mrm.1910310307 8057799

[21] Friston KJ, Williams S, Howard R, Frackowiak RSJ, Turner R. Movement-Related effects in fMRI time-series. Magn Reson Med [Internet]. 1996 3 3;35(3):346–55. Available from: http://doi.wiley.com/10.1002/mrm.1910350312 10.1002/mrm.1910350312 8699946

[22] Arnold S, Vogler M, Hinds O, Hamm M, Pfeuffer J, Triantafyllou C. Evaluation of EPI Geometric Distortion Correction using Phase Labeling for Additional Coordinate Encoding (PLACE). 2009;57(4):4633.

[23] Vogler M, Arnold S, Hinds O, Pfeuffer J, Whitfield-gabrieli S, Triantafyllou C. Inline Distortion Correction for Echo-Planar fMRI. Proc 14th Annu Meet Organ Hum Brain Mapp. 2008;

[24] Raj D, Anderson a W, Gore JC. Respiratory effects in human functional magnetic resonance imaging due to bulk susceptibility changes. Phys Med Biol [Internet]. 2001 12;46(12):3331–40. Available from: http://www.ncbi.nlm.nih.gov/pubmed/11768509 10.1088/0031-9155/46/12/318 11768509

[25] Pfeuffer J, Wang D, Triantafyllou C. Dynamic Phase Echo-Planar Imaging—Detection and Correction of Dynamic Off-Resonance 1. Proc Intl Soc Mag Reson Med. 2011;19(1994):4577.

[26] Zeller M, Kraus P, Müller A, Bley T a, Köstler H. Respiration impacts phase difference-based field maps in echo planar imaging. Magn Reson Med [Internet]. 2013 9 9 [cited 2013 Oct 23];00:1–6. Available from: http://www.ncbi.nlm.nih.gov/pubmed/24018714 10.1002/mrm.24938 24018714

[27] Pfeuffer J, Van de Moortele P-F, Ugurbil K, Hu X, Glover GH. Correction of physiologically induced global off-resonance effects in dynamic echo-planar and spiral functional imaging. Magn Reson Med [Internet]. 2002 2 [cited 2013 Oct 25];47(2):344–53. Available from: http://www.ncbi.nlm.nih.gov/pubmed/11810679 10.1002/mrm.10065 11810679

[28] Zeller M, Müller A, Hahn D, Köstler H. Phase-labeled reference EPI for frequency-segmented inhomogeneity corrections (PREFICS). Magn Reson Med [Internet]. 2013 3 29 [cited 2013 Oct 25];000:1–6. Available from: http://www.ncbi.nlm.nih.gov/pubmed/23554070 10.1002/mrm.24737 23554070

[29] Chavez S, Ramsay E, Haidar M, Xiang Q-S, Stanisz G. Distortion correction of multi-coil diffusion-weighted EPI using the phase-based method: PLACE. Proc Intl Soc Mag Reson Med. 2010;5064.

[30] Heid O. Method for the phase correction of nuclear magnetic resonance signals. US Pat 6043651A. 2000;

[31] Friedman L, Glover GH. Report on a multicenter fMRI quality assurance protocol. J Magn Reson Imaging [Internet]. 2006 6 [cited 2015 Feb 10];23(6):827–39. Available from: http://www.ncbi.nlm.nih.gov/pubmed/16649196 10.1002/jmri.20583 16649196

[32] Jenkinson M. Fast, automated, N-dimensional phase-unwrapping algorithm. Magn Reson Med [Internet]. 2003 1 [cited 2014 Jan 31];49(1):193–7. Available from: http://www.ncbi.nlm.nih.gov/pubmed/12509838 10.1002/mrm.10354 12509838

[33] Cox RW. AFNI: software for analysis and visualization of functional magnetic resonance neuroimages. Comput Biomed Res [Internet]. 1996 6;29(3):162–73. Available from: http://www.ncbi.nlm.nih.gov/pubmed/8812068 10.1006/cbmr.1996.0014 8812068

[34] McCullagh P. Generalized linear models. Eur J Oper Res. 1984;16:285–92.

[35] Benjamini Yoav and H Y. Controlling the false discovery rate: a practical and powerful approach to multiple testing. Journal of the Royal Statistical Society. Series B (Methodological); 1995 p. 289–300.

[36] Hagberg GE, Bianciardi M, Brainovich V, Cassarà AM, Maraviglia B. The effect of physiological noise in phase functional magnetic resonance imaging: from blood oxygen level-dependent effects to direct detection of neuronal currents. Magn Reson Imaging [Internet]. 2008 9 [cited 2013 Nov 12];26(7):1026–40. Available from: http://www.ncbi.nlm.nih.gov/pubmed/18479875 10.1016/j.mri.2008.01.010 18479875

[37] Van de Moortele P-F, Pfeuffer J, Glover GH, Ugurbil K, Hu X. Respiration-induced B0 fluctuations and their spatial distribution in the human brain at 7 Tesla. Magn Reson Med [Internet]. 2002 5 [cited 2013 Oct 25];47(5):888–95. Available from: http://www.ncbi.nlm.nih.gov/pubmed/11979567 10.1002/mrm.10145 11979567

[38] Cheng H, Li Y. Respiratory noise correction using phase information. Magn Reson Imaging [Internet]. Elsevier Inc.; 2010 5 [cited 2015 May 29];28(4):574–82. Available from: http://www.ncbi.nlm.nih.gov/pubmed/20096522 10.1016/j.mri.2009.12.014 20096522

[39] Rotenberg D, Chiew M, Ranieri S, Tam F, Chopra R, Graham SJ. Real-time correction by optical tracking with integrated geometric distortion correction for reducing motion artifacts in functional MRI. Magn Reson Med [Internet]. 2013 3 1 [cited 2013 Oct 25];69(3):734–48. Available from: http://www.ncbi.nlm.nih.gov/pubmed/22585554 10.1002/mrm.24309 22585554

[40] Thesen S, Heid O, Mueller E, Schad LR. Prospective acquisition correction for head motion with image-based tracking for real-time fMRI. Magn Reson Med [Internet]. 2000 9;44(3):457–65. Available from: http://www.ncbi.nlm.nih.gov/pubmed/10975899 10.1002/1522-2594(200009)44:3<457::aid-mrm17>3.0.co;2-r 10975899

[41] Zaitsev M, Dold C, Sakas G, Hennig J, Speck O. Magnetic resonance imaging of freely moving objects: prospective real-time motion correction using an external optical motion tracking system. Neuroimage [Internet]. 2006 7 1 [cited 2013 Oct 25];31(3):1038–50. Available from: http://www.ncbi.nlm.nih.gov/pubmed/16600642 10.1016/j.neuroimage.2006.01.039 16600642

[42] Aksoy M, Forman C, Straka M, Skare S, Holdsworth S, Hornegger J, et al Real-time optical motion correction for diffusion tensor imaging. Magn Reson Med [Internet]. 2011 8 [cited 2013 Oct 25];66(2):366–78. Available from: http://www.pubmedcentral.nih.gov/articlerender.fcgi?artid=3139706&tool=pmcentrez&rendertype=abstract 10.1002/mrm.22787 PMC3139706 21432898

[43] Speck O, Hennig J, Zaitsev M. Prospective real-time slice-by-slice motion correction for fMRI in freely moving subjects. MAGMA [Internet]. 2006 5 [cited 2013 Oct 25];19(2):55–61. Available from: http://www.ncbi.nlm.nih.gov/pubmed/16779560 10.1007/s10334-006-0027-1 16779560

[44] Glover GH, Li T, Ress D. Image-Based Method for Retrospective Correction of Physiological Motion Effects in fMRI: RETROICOR. Magn Reson Med. 2000;167(March):162–7. 10893535 10.1002/1522-2594(200007)44:1<162::aid-mrm23>3.0.co;2-e

[45] Faraji-Dana Z, Tam F, Chen JJ, Graham S. “Importance of physiological noise correction for PLACE Distortion Correction in EPI—based fMRI.”Proc 21st Annu Meet Organ Hum Brain Mapp. 2015;

[46] Hagberg GE, Bianciardi M, Brainovich V, Cassara AM, Maraviglia B. Phase stability in fMRI time series: effect of noise regression, off-resonance correction and spatial filtering techniques. Neuroimage [Internet]. Elsevier Inc.; 2012 2 15 [cited 2014 May 2];59(4):3748–61. Available from: http://www.ncbi.nlm.nih.gov/pubmed/22079450 10.1016/j.neuroimage.2011.10.095 22079450

